# Direct Observation of Confinement Effects of Semiconducting Polymers in Polymer Blend Electronic Systems

**DOI:** 10.1002/advs.202100332

**Published:** 2021-05-14

**Authors:** Byoungwook Park, Hongkyu Kang, Yeon Hee Ha, Jehan Kim, Jong‐Hoon Lee, Kilho Yu, Sooncheol Kwon, Soo‐Young Jang, Seok Kim, Soyeong Jeong, Soonil Hong, Seunghwan Byun, Soon‐Ki Kwon, Yun‐Hi Kim, Kwanghee Lee

**Affiliations:** ^1^ Heeger Center for Advanced Materials Gwangju Institute of Science and Technology Gwangju 61005 Republic of Korea; ^2^ Research Institute for Solar and Sustainable Energies Gwangju Institute of Science and Technology Gwangju 61005 Republic of Korea; ^3^ Department of Chemistry and Research Institute for Green Energy Convergence Technology Gyeongsang National University Jinju 52828 Republic of Korea; ^4^ Pohang Accelerator Laboratory Pohang University of Science and Technology Pohang 790‐784 Republic of Korea; ^5^ School of Materials Science and Engineering Gwangju Institute of Science and Technology Gwangju 61005 Republic of Korea; ^6^ Department of Chemistry Gwangju Institute of Science and Technology Gwangju 61005 Republic of Korea; ^7^ Department of Materials Engineering and Convergence Technology and ERI Gyeongsang National University Jinju 52828 Republic of Korea

**Keywords:** organic field‐effect transistors, physical confinement, polymer blends, polymer nano‐networks, transparent polymer semiconductors

## Abstract

The advent of special types of polymeric semiconductors, known as “polymer blends,” presents new opportunities for the development of next‐generation electronics based on these semiconductors' versatile functionalities in device applications. Although these polymer blends contain semiconducting polymers (SPs) mixed with a considerably high content of insulating polymers, few of these blends unexpectedly yield much higher charge carrier mobilities than those of pure SPs. However, the origin of such an enhancement has remained unclear owing to a lack of cases exhibiting definite improvements in charge carrier mobility, and the limited knowledge concerning the underlying mechanism thereof. In this study, the morphological changes and internal nanostructures of polymer blends based on various SP types with different intermolecular interactions in an insulating polystyrene matrix are investigated. Through this investigation, the physical confinement of donor–acceptor type SP chains in a continuous nanoscale network structure surrounded by polystyrenes is shown to induce structural ordering with more straight edge‐on stacked SP chains. Hereby, high‐performance and transparent organic field‐effect transistors with a hole mobility of ≈5.4 cm^2^ V^–1^ s^–1^ and an average transmittance exceeding 72% in the visible range are achieved.

## Graphical abstract

The confinement effect of semiconducting polymer (SP) is a core factor directly influencing the charge transport properties of polymer blend electronic systems. This study generalizes that the physical confinement of donor–acceptor type SP can induce continuously connected SP network in insulating polymer matrix and the structural ordering with more straight edge‐on stacked SP chains enable an efficient charge transport.

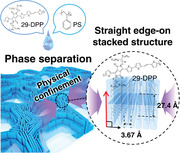

## 1 Introduction

Polymer blend electronics, which are based on a composite of a semiconducting polymer (SP) and insulating polymer (IP), have advanced rapidly and garnered significant interest because of their attractive potential in various applications such as, transparent electronic,^[^ ^1^, ^2^, ^3^ ^]^ stretchable/flexible electronic,^[^ ^4^, ^5^, ^6^, ^7^, ^8^, ^9^, ^10^, ^11^ ^]^ and self‐healing/biodegradable electronic^[^ ^12^, ^13^, ^14^, ^15^, ^16^ ^]^ devices. The key merit of the polymer blend approach is the fact that this approach enables integration of specific physical properties of SPs and IPs without sophisticated material synthesis being required, while maintaining the semiconducting properties of SPs.^[^ ^17^, ^18^, ^19^ ^]^ Pioneering studies on SP:IP blends have demonstrated that this simple blending method can achieve various beneficial functionalities, including patterning processability,^[^ ^20^ ^]^ mechanical stretchability,^[^ ^4^, ^5^, ^6^, ^8^, ^9^, ^10^, ^11^ ^]^ self‐healability,^[^ ^12^, ^13^ ^]^ air stability,^[^ ^21^, ^22^ ^]^ thermal stability,^[^ ^23^ ^]^ and excellent optical transparency.^[^ ^1^, ^2^, ^21^ ^]^ Furthermore, in addition to the studies exploring the integration of the physical properties, a few notable works have demonstrated that despite containing a significant amount of IPs, SP:IP blend systems exhibit charge carrier mobilities that are comparable or even superior to those of pure SPs, along with newly arising polymer blend characteristics.^[^ ^2^, ^10^, ^19^, ^20^, ^23^, ^24^, ^25^, ^26^, ^27^, ^28^, ^29^, ^30^, ^31^, ^32^, ^33^, ^34^ ^]^

As a basis for this synergistic effect in the SP:IP blend systems, so‐called “confinement effect” of SPs, which arises from phase separation of the SP:IP blends placing SP chains into well‐defined condensed domains with specific morphologies, has been generally considered.^[^ ^5^, ^35^, ^36^, ^37^, ^38^, ^39^ ^]^ This is because the confinement of SPs within an IP matrix in the SP:IP blends enables the incorporation of the material properties of both SPs and IPs, owing to the stable coexistence of the physically separated SP and IP phases. In addition, a continuously connected network of SPs is generated through the confinement‐induced interchain coupling of SP chains, which improves even the charge transport characteristics.^[^ ^2^, ^32^, ^34^ ^]^ However, despite these desirable features, the combinational basis for why the incorporation of an SP into an IP can sometimes elicit the confinement effect of SPs has so far not been clarified. In addition, because of the intrinsic structural congestion of polymer blend systems and the lack of cases specifically showing the confinement effect of SPs in the IP matrix, it has been rather difficult to develop a general understanding on the conformational ordering and orientational alignment of confined SPs within the IP matrix, as a core factor directly influencing the charge transport. Consequently, the insight into synergistic polymer blend systems ensuring a specific enhancement in their charge carrier mobilities has not yet been clearly understood. Thus, to progress toward the achievement of ideal polymer blend systems that maximize the multifunctional advantages, it is necessary to clarify the confinement effect of SPs and devise an effective strategy for designing multi‐talented polymer blend composites. Herein, we systematically investigated the confinement effect depending on the type of intermolecular interaction of the SPs in the IP of polystyrene (PS) matrices, and we developed synergistic SP:PS polymer blend systems that exhibit an improvement in the field‐effect hole mobilities by 3–4 times (*μ*_h,max_ approaching 10 cm^2^ V^–1^ s^–1^) along with excellent optical transmittance (*T*) exceeding 95% in the visible range. Through detailed morphological investigations via transmission electron microscopy (TEM) and atomic force microscopy (AFM) for various blend films with different SP:PS ratios, we found that the strong intermolecular interaction of SPs based on molecular planarity and polarity can induce the formation of a confined network structure of SPs in the polymer blend composites. In addition, the combination of temperature‐dependent charge carrier mobility analysis and grazing incident‐wide angle X‐ray spectroscopy (GIWAXS) indicated the nanostructure of confined SPs that exhibits a straight edge‐on stacking structure in the blends. We believe that this ordered structure of the SP chains in the blend systems is responsible for the substantially enhanced mobilities with much lower activation energies to facilitate charge movement along the SP chains, as compared to those of pure SP films. Finally, with the transparent gate/source/drain electrodes, we successfully fabricated all‐transparent organic field‐effect transistors (OFETs) having an overall device transmittance exceeding 72% and *μ*_h_ of 5.4 cm^2^ V^–1^ s^–1^ by using the best polymer blend systems.

## 2 Results and Discussion

**Figure 1:**
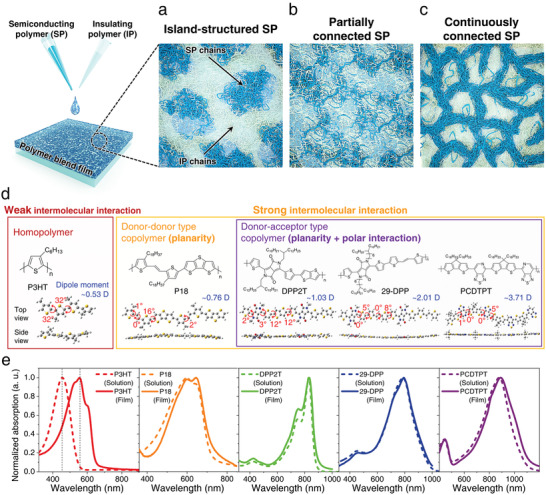
Various types of SPs with different intermolecular interactions. Schematic illustrations of the representative structural morphologies of SP:IP blend films: a) Island‐structured SP, b) partially connected SP, and c) continuously connected SP. d) DFT‐optimized molecular models and calculated dipole moments of dimer (two repeating units) of P3HT, P18, DPP2T, 29‐DPP, and PCDTPT (simulation model: B3LYP/6‐311G**). e) Normalized absorption spectra of P3HT, P18, DPP2T, 29‐DPP, and PCDTPT in solution and on thin film.

The evaluation of the SP dispersion in the IP matrix is important because the physical and charge transport properties are strongly related to the morphologies obtained. Depending on the dispersion of the SP chains, three possible types of polymer blend morphologies exist in SP:IP blend films (**Figure** **1**a): Island‐structured SP, partially connected SP, and continuously connected SP morphology. When each SP domain is completely physically separated, the island‐structured SP morphology is obtained (Figure 1a), resulting in poor charge transport properties. A moderate interchain coupling between each SP domain could facilitate the formation of a partially connected SP morphology, whose charge transport properties are degraded in proportion to the discontinuity of the charge transport pathway. (Figure 1b) However, if the strong connection between SP domains is presented with the interchain coupling of SP chains, a continuously connected SP morphology can be achieved, resulting in the most efficient charge transport characteristics of SP:IP blend systems because such a morphology ensures undisturbed charge transport. Therefore, to obtain a better understanding of the formation of continuously connected SP morphologies and their charge transport characteristics, we designed a systematic investigation of SP:IP blend composites depending on the type of intermolecular interaction of SPs.

The molecular structures of various SPs are depicted in Figure 1d. By employing a variety of SPs, including poly(3‐hexylthiophene) (P3HT), poly[(E)‐1,2‐(3,3′‐dioctadecyl‐2,2′‐dithienyl)ethylene‐alt‐dithieno‐(3,2‐b:2′,3′‐d)thiophene] (P18),^[^ ^40^ ^]^ DT‐PDPP2T‐TT (DPP2T),^[^ ^41^ ^]^ poly[2,5‐bis(2‐decylnonadecyl)pyrrolo[3,4‐c]pyrrole‐1,4‐(2H,5H)‐dione‐(E)‐1,2‐di(2,2′‐bithioph en‐5yl)ethene] (29‐DPP),^[^ ^42^, ^43^ ^]^ and regioregular poly[4‐(4,4‐dihexadecyl‐4H‐cyclopenta[1,2‐b:5,4‐b′]dithiophen‐2yl)‐alt‐[1,2,5]thiadiazolo[3,4‐c]pyridine] (PCDTPT),^[^ ^44^ ^]^ we intended to clarify the dependence of intermolecular interactions of SPs on the connectivity of SP domains within the IP matrix. First, to evaluate the intermolecular interaction of SPs based on the planarity and polarity of the polymer back bone, we calculated the dihedral angle and dipole moments of each SP by simulating the molecular architectures from the dimer conformation of five SPs via the B3LYP/6‐311G** density functional theory method. For conciseness, the simulations were performed with the dimers of each polymer having methyl side chains in place of alkyl chains. As a result, P18, DPP2T, 29‐DPP, and PCDTPT polymers exhibited a planar conformation of the dimer units, as shown in Figure 1a (side view, bottom), while P3HT had a lower planarity of the backbone because of steric hindrance between adjacent alkylated thiophenes. Generally, the high planarity of the conjugated backbone of SPs promotes delocalization of charge carriers and intermolecular *π*‐interactions. On the other hand, regarding the results pertaining to the dipole moments of SPs, each SP presents the calculated dipole moment value of 0.53 debye (D) (P3HT), 0.76 D (P18), 1.03 D (DPP2T), 2.01 D (29‐DPP), and 3.71 D (PCDTPT). On the basis of these results, it can be speculated that DPP2T, 29‐DPP, and PCDTPT can additionally develop a strong polar intermolecular interaction, when compared to P3HT (homopolymer) and P18 (D‐D copolymer), because they possess relatively large dipole moments in the polymer backbone with a polarity difference between the donor–acceptor (D–A) units.^[^ ^45^, ^46^, ^47^, ^48^, ^49^ ^]^ In addition, to identify the aggregation strength of the SPs, we checked their UV–vis absorption spectra considering both the solution and film states. As shown in Figure 1b, P18, DPP2T, 29‐DPP, and PCDTPT polymers demonstrated almost negligible bathochromic shifts of absorption peaks between solutions and films, which implies that P18, DPP2T, 29‐DPP, and PCDTPT polymers undergo pre‐aggregation even in their solution states with strong intermolecular interactions, whereas the absorption peak of the P3HT solution (≈456 nm) drastically shifted to 555 nm in the film state.

We selected PS as the counterpart IP because of its nonpolar property that facilitates a low molecular interaction with SPs and prevents the degradation of the charge carrier mobility caused by a dipolar effect arising from the existence of the permanent dipole moments of blended polymers, in contrast to the case of other relatively polar IPs.^[^ ^50^ ^]^ For the preparation of the polymer blend solutions, each of the SP and PS solutions was separately prepared in halogenated hydrocarbon‐based solvents, following which the SP solutions were diluted in the PS solution with different SP:IP ratios, in which the total concentrations of the polymer blends were constant.

**Figure 2:**
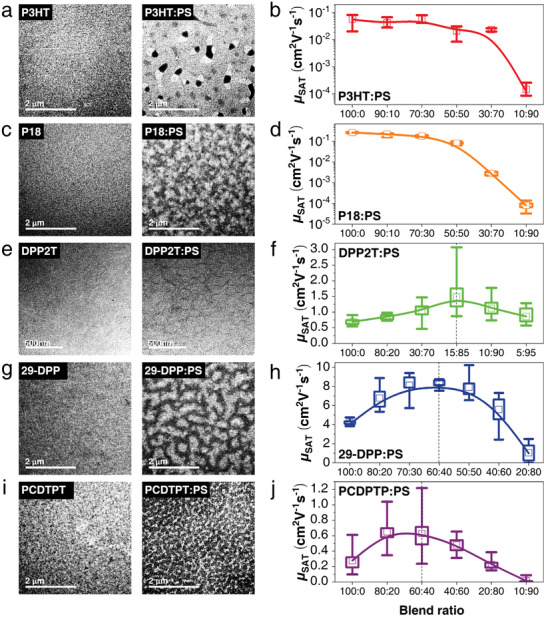
Correlation between morphological properties and charge transport characteristics of polymer blend composites with various type of SPs and PSs. TEM images of the spin‐cast thin films of pure SPs and SP:PS blends. a) P3HT and P3HT:PS blend; c) P18 and P18:PS blend; e) DPP2T and DPP2T:PS; g) 29‐DPP and 29‐DPP:PS blend; i) PCDTPT and PCDTPT:PS blend films. Field‐effect hole mobility in SP:PS blend OFET devices with various blend ratios; b) P3HT:PS blend; d) P18:PS blend; f) DPP2T:PS; h) 29‐DPP:PS blend; j) PCDTPT:PS blend films.

To investigate the characteristic morphological changes of various types of SP:IP blend composites, the nanomorphology images of pure SP and SP:IP blend films were recorded via TEM and AFM and analyzed (**Figure** **2**a,c,e,g,i and Figure S2, Supporting Information). In all the pure SP films, only a regular morphology with uniformly dispersed phases was observed without any remarkable features. On the other hand, the SP:IP blend films showed distinctly different surface morphologies when compared to those of the pure SP films, but the phase separation of the SP and IP was significantly distinguished depending on the intermolecular interaction of the SPs. The P3HT:PS blend films exhibited a film formation in which the PS aggregation was separately embedded in the P3HT film; however, the PS aggregation displayed continuous interconnection with an increase in the ratio of PS, resulting in the formation of island‐structured P3HT morphologies. Moreover, the P18:PS blend films showed morphologies in which the stacking of P18 was gradually disassembled with the increase in the proportion of PSs. However, in the DPP2T:PS, 29‐DPP:PS, and PCDTPT:PS blend films, it was observed that the D–A type SPs certainly achieved lateral phase separation from the PS, and the confined D–A type SPs formed a 2D network structure in inert PS matrices, while there is no significant vertical phase separation of the films due to a small difference in hydrophilicity between the SP and PS materials (Figure S3 and Table S1, Supporting Information).^[^ ^24^, ^51^, ^52^ ^]^ In accordance with previous studies on conventional IP:IP polymer blends, it can be assumed that the physical effect of confinement may be attributed to the SP:PS blend morphologies.^[^ ^53^, ^54^ ^]^ Moreover, a higher degree of chain‐chain interactions or backbone planarity could be deduced from an increase in the ratio between the 0‐0/0‐1 peaks of the DPP2T:PS, 29‐DPP:PS, and PCDTPT:PS blend films, as compared with the neat DPP2T, 29‐DPP, and PCDTPT films (Figure S4, Supporting Information).^[^ ^10^, ^11^, ^32^, ^34^ ^]^

Base on the direct contrast in the nanomorphologies of the blend films, to identify fundamentally different charge transport characteristics in each SP:PS blend systems, we explored the charge transport characteristics of polymer blend films with various SP:PS blend ratios by fabricating the OFETs (Figure 2b,d,f,h and Figure S5, Tables S2 and S3, Supporting Information). For the practical comparison of SP:PS blend films with various blend ratios, the saturation hole mobilities (*μ*_h)_ of SP:PS blend films was extracted assuming the SP:PS blend film itself as a semiconducting layer. Each of the pure P3HT, P18, DPP2T, 29‐DPP, and PCDTPT films (SP:PS ratio = 100:0) exhibits saturation hole mobilities as high as 7.4 × 10^–2^, 0.27, 0.91, 4.7, and 0.61 cm^2^ V^–1^ s^–1^ (average 5.3 × 10^–2^, 0.26, 0.69, 4.2, and 0.28 cm^2^ V^–1^ s^–1^), respectively, which are similar to previously reported values.^[^ ^40^, ^41^, ^42^, ^43^, ^44^ ^]^ In contrast, while the *μ*_h_ values of the P3HT:PS and P18:PS blend films gradually deteriorated as the proportion of inert PS increase, it was found that despite the lesser content of SPs than the pure SP films, the *μ*_h_ values of the DPP2T, 29‐DPP, and PCDTPT blend films are significantly improved by three or four times; *μ*_h_,_max_ is 3.1 cm^2^ V^–1^ s^–1^ for DPP2T, 10 cm^2^ V^–1^ s^–1^ for 29‐DPP:PS, and 1.2 cm^2^ V^–1^ s^–1^ for PCDTPT:PS (1.5, 8.3, and 0.63 cm^2^ V^–1^ s^–1^, respectively).

**Figure 3:**
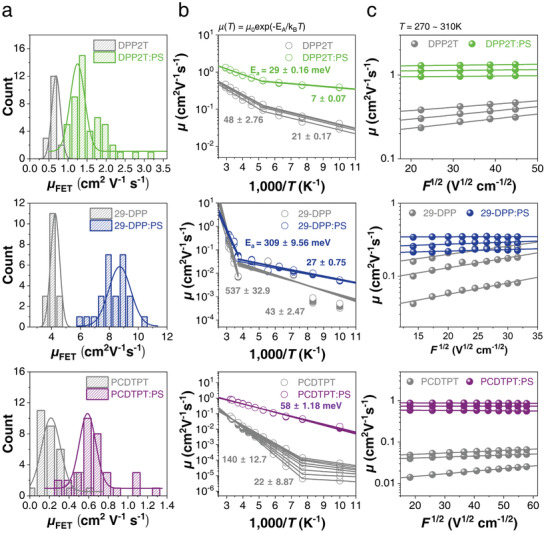
Temperature (*T*) and electric‐field (*F*) dependence characteristics of pure SPs and SP:PS blend films. a) Hole mobility histograms of pure SPs and SP:PS blend OFET devices with various blend ratios. b) Arrhenius plot of the temperature‐dependent linear hole mobility of pure SPs and SP:PS blend OFETs for various drain biases (DPP2T and DPP2T:PS = −2 to −8 V; 29‐DPP, 29‐DPP:PS, PCDTPT, and PCDTPT:PS = −2 to −10 V). c) Plot of the linear hole mobility as a function of applied electric field (*F* ^1/2^) for pure SPs and SP:PS blend OFETs at *T* = 270–310 K.

To obtain a deeper understanding of the charge transport mechanism in the DPP2T:PS, 29‐DPP:PS, and PCDTPT:PS blend films, we measured and compared the temperature‐dependent FET characteristics based on the pure SP and SP:PS blend films. **Figure** **3**a exhibits the Arrhenius plot of the temperature dependence of linear mobility (*μ*_lin_) of the pure SP‐based FETs and SP:PS blend‐based FETs under varying drain‐source voltage (*V*_DS_). Using the Arrhenius relation,

(1) $$\mu \left(T\right)=\mu_{0}\exp\left(-E_{A}/k_{B}T\right)$$

where *μ*_0_ is the charge carrier mobility in the trap‐free states, *E*_A_ is the Arrhenius‐type activation energy, *k*_B_ is the Boltzmann constant, and *T* is the absolute temperature, we calculated the *E*_A_ values.^[^ ^55^, ^56^, ^57^, ^58^ ^]^ In most FETs except for the one using the PCDTPT:PS blend film, the plot of temperature dependence of mobility exhibited two activation regimes distinguished by a critical transition temperature, which is the minimum thermal energy essential for charge transport between aggregated and amorphous SP regions.^[^ ^59^ ^]^ In both low‐temperature (intra‐chain transport dominant) and high‐temperature (intra‐chain transport and inter‐chain transport) regimes, all of the three SP:PS blend systems show significantly reduced activation energies than the pure SP system, signifying efficient charge transport between localized states within the 2‐D confined network structure of SPs. Furthermore, the SP:PS blends have only negligible electric field dependence of mobility in the temperature range from 270 K to 310 K, whereas the pure SP films exhibit linear increase in the plot showing electric field dependence of mobility (Figure 3c).^[^ ^60^ ^]^ Overall, these results indicate that disorder‐limited charge transport is reduced in the polymer blends presumably due to the formation of the highly ordered crystalline SP bundles within the 2‐D confined network structure of SPs. In addition, in the case of the PCDTPT:PS blend film, the deviation of charge carrier mobility as a function of electric field at low temperature was significantly reduced when compared to that of the pure PCDTPT film, which indicates the reduced trap sites of PCDCPT:PS blend film. Although it was not possible to confirm the charge transport characteristics at extremely low temperature below 100 K, therefore, it could be surmised that the critical transition temperature of the PCDTPT:PS blend film would shift to a lower temperature than the pure PCDTPT films as a result of the reduction of thermal energy required for inter‐chain transport (Figure S6, Supporting Information).

**Figure 4:**
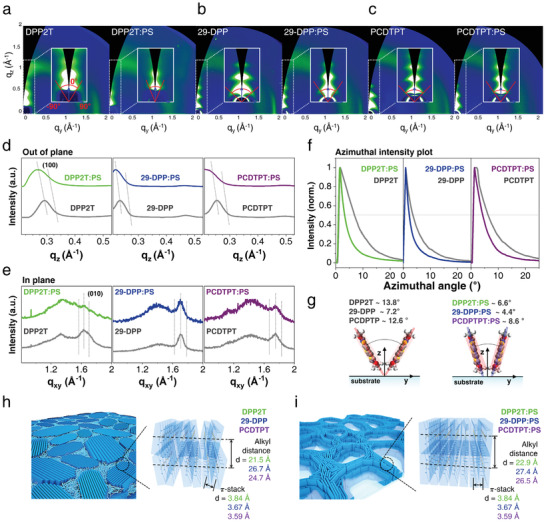
GIWAXS structure characterization of pure SPs and SP:PS blend films. 2D GIWAXS images recorded for a) DPP2T, DPP2T:PS blend, b) 29‐DPP, 29‐DPP:PS blend, c) PCDTPT and PCDTPT:PS blend films. The corresponding 1D scattering patterns: 1D intensity profiles of the pure SPs and SP:PS blend films; d) the out of plane angle; e) the in‐plane angle; and f) the azimuthal angle of the (100) peak direction. g) Orientational alignment of SP backbones in the pure SPs and SP:PS blend films. Schematic illustrations of the structural morphologies and molecular packing structures of h) pure SPs and i) SP:PS blends.

Direct evidence of the molecular arrangement and the crystallinity within the SP bundles can be obtained from the GIWAXS measurements (**Figure** **4**). The diffraction patterns of the pure SP and SP:PS blend films for DPP2T, 29‐DPP, and PCDTPT show clear preferences for the edge‐on orientation of the polymer backbones on the substrate surfaces with both the (100) peak in out of plane and (010) peak in in‐plane direction (Figure 4a–c). In particular, despite the high contents of the amorphous PS in the blends, a highly ordered lamellar structure that extends the out‐of‐plane diffraction to a value of (400) can be observed in the DPP2T:PS, 29‐DPP:PS, and PCDTPT:PS blend films. This observation is similar to those observed for other high‐mobility pure SPs, in which the edge‐on structures of SPs allows efficient in‐plane transport both along the polymer backbone and the direction of *π*‐*π* stacking.^[^ ^61^, ^62^, ^63^ ^]^ In the GIWAXS pattern of the pure SP films, the (100) peaks along the vertical axis at *q*_z, DPP2T_ = 0.292 Å^–1^, *q*_z, 29‐DPP_ = 0.235 Å^–1^, and *q*_z, PCDTPT_ = 0.254 Å^–1^ correspond to the lamellar stacking at 21.5 Å for DPP2T, 26.7 Å for 29‐DPP, and 24.7 Å for PCDTPT (Figure 4d), respectively. In addition, the in‐plane diffraction signals originating from (010) diffraction at *q*_y, DPP2T_ = 1.63 Å^–1^, *q*_y, 29‐DPP_ = 1.71 Å^–1^, and *q*_y, PCDTPT_ = 1.75 Å^–1^ provide information on the *π*‐*π* stacking distances of 3.84 Å for DPP2T, 3.67 Å for 29‐DPP, and 3.59 Å for PCDTPT (Figure 4e), respectively. On the other hand, in the GIWAXS patterns of the SP:PS blend films, the lamellar stacking distances in the out of plane direction slightly increase as the peaks shift to *q*_z, DPP2T_ = 0.274 Å^–1^ (*d* = 22.9 Å), *q*_z, 29‐DPP_ = 0.230 Å^–1^ (*d* = 27.4 Å), and *q*_z, PCDTPT_ = 0.237 Å^–1^ (*d* = 26.5 Å), while the *π*‐*π* stacking distance of 3.84, 3.67, and 3.59 Å in the in‐plane direction remained the same as in the pure SP films (Figure 4d,e).

However, observation of the increased lamellar stacking distances and no change in the *π*‐*π* stacking distances in the SP:PS blend films do not support the dramatic enhancement of the field‐effect mobilities in the blend films. We anticipate that the physical confinement in the polymer blends induces molecular ordering of the SP chains in the blend systems as well as formation of 2‐D confined network structure of SPs. To obtain further insight into molecular stacking, therefore, the azimuthal angle plot and full width at half‐maximum (FWHM) of the (100) peaks are subsequently analyzed because the degree of preferential z‐axis alignment of polymer chains can be characterized by the FWHM of out of plane peaks in the azimuthal angle plot of GIWAXS patterns (Figure 4f).^[^ ^34^ ^]^ In the edge‐on stacking structure, not only the (010) peak in the inplane direction and the (100) peak in the out of plane direction have the same structural origin, but the (010) peak in the inplane direction allows to evaluate the orientational alignment of the SP back bones without interference from overlapping with the amorphous halo peak of SiO_2_ used for the GIWAXS analysis (Figure S7, Supporting Information).^[^ ^34^, ^64^ ^]^ By extracting 1D profiles from 2D GIWAXS patterns, the FWHM of the pure DPP2T, 29‐DPP, and PCDTPT films for (100) peak are 13.8°, 7.2°, and 12.6°, respectively, whereas for the DPP2T:PS, 29‐DPP:PS, and PCDTPT:PS blend films, the FWHM of the azimuthal angles are 6.6°, 4.4°, and 8.6°, respectively (Figure 4g). These results indicate that the SP:PS blend films are more straightly stacked than the pure SP films; the straight interdigitation of alkyl chains would cause an increase in the alkyl chain distance along the z‐axis (Figure 4h,i). The straight edge‐on stacked SPs can support an efficient charge transport pathway because the regular alignment of the SP chains can induce more extended orbital overlapping along the polymer backbone as well as small trap sites arising from the reduction of free volume between polymer chains. Therefore, it is noteworthy that the straight edge‐on alignment of SPs can be effectively induced by blending SPs with PS, which can be achieved via a simple spin‐coating method without any complicated film deposition process.

**Figure 5:**
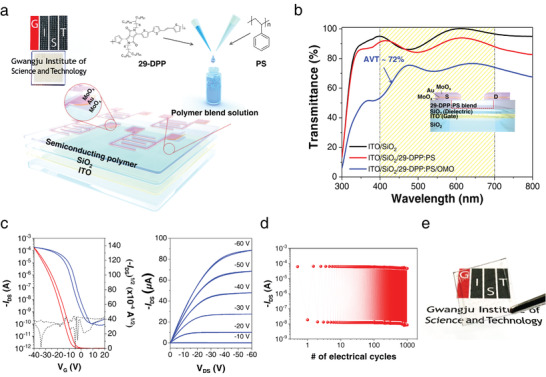
All transparent 29‐DPP:PS blend OFETs with transparent electrodes. a) Schematic of the structure of all transparent 29‐DPP:PS blend OFETs. b) Optical transmittance spectra of 29‐DPP:PS blend OFETs measured in different layering conditions. c) Transfer and output characteristics of the 29‐DPP:PS blend OFETs. d) The cycling voltage test of 29‐DPP:PS blend OFETs at *V*_d_ = −60 and *V*_g_ = −40 V. e) Optical image of all transparent 29‐DPP:PS blend OFET device.

To evaluate the merit of superior carrier mobility and high visible transparency of the SP:PS blend film, we fabricated transparent FETs with a bottom gate‐top contact configuration by using a semitransparent molybdenum oxide/Au/molybdenum (5 nm/4 nm/30 nm) oxide (OMO) electrode as a top source‐drain electrode and indium tin oxide (ITO)/silicon dioxide (SiO_2_) as bottom gate electrode and dielectric layer (**Figure** **5**a and Figures S9 and S10, Supporting Information). Figure 5b shows the optical transmittance spectra of the 29‐DPP:PS blend‐based FET obtained with different layering conditions. As can be seen in Figure 5b, the overall visible transmittance of the FET is about 72%, even including that of the semitransparent OMO source/drain electrodes (Figure 5e and Figure S8 and Table S4, Supporting Information). The transparent 29‐DPP:PS polymer blend transistors exhibit a maximum mobility, *μ*_h,max_, ≈5.4 cm^2^ V^–1^ s^–1^, threshold voltage ≈−4.4 V, and on/off ratio ≈10^6^ (Figure 5c). Furthermore, our all‐transparent polymer blend transistors have an excellent operating stability when considering that the retention time is sufficient for long‐term data storage against bias stress; after 1000 switching cycles, the on/off current remains almost constant with a slight decrease at the end of the cycle (Figure 5d). To the best of our knowledge, these results present the best performance in terms of high device transmittance and charge carrier mobility in polymer FETs, without involving any uncontrollable and complex process. We believe that, in addition to the SP:IP blend technique, the development of novel transparent electrodes for the fabrication of high‐quality FETs could synergistically lead to further improvement in the transparency of FETs.

## 3 Conclusion

In conclusion, the confinement effect allowing for the formation of a continuously connected phase of SPs is one of the most important factors determining the charge transport characteristics of SP:IP blend systems. Our work presents a systematic approach to investigate the confinement effect depending on the type of intermolecular interactions of SPs in SP:IP blend films. On the basis of a comparison between the physical and electrical characteristics of various types of SP:IP blend films, we found that the D–A type SP capable of polar intermolecular interaction spontaneously developed a confinement‐induced straight edge‐on stacked structure in the inert PS matrices, and it facilitated more efficient charge transport with reduced activation energies and low densities of trap states. On the basis of the high transparency and improved charge carrier mobility features of the blend films, transparent OFETs with an overall device transmittance of ≈72% and a charge mobility of ≈5.4 cm^2^ V^–1^ s^–1^ were fabricated, showing excellent operating stability. Overall, our results provide new insights into the molecular structure conformation and charge transport properties of polymer blends, which are potential materials for use in next‐generation display technologies.

## Conflict of Interest

The authors declare no conflict of interest.

## Supporting information

Supporting Information

## Data Availability

Research data are not shared.
