# Supplementary material for: Direct Observation of Confinement Effects of Semiconducting Polymers in Polymer Blend Electronic Systems
Source: Adv Sci (Weinh). 2021 May 14;8(14):2100332. doi: 10.1002/advs.202100332 (PMC8292904; doi:10.1002/advs.202100332)
Supplement: Supplementary file 1 — Supporting Information [file ADVS-8-2100332-s001.pdf]

© 2021 Wiley-VCH GmbH

ADVANCED SCIENCE
Open Access

Supporting Information

for *Adv. Sci.,* DOI: 10.1002/advs.202100332

Direct Observation of Confinement Effects of Semiconducting Polymers in Polymer Blend Electronic Systems

Byoungwook Park, Hongkyu Kang, Yeon Hee Ha, Jehan Kim, Jong-Hoon Lee, Kilho Yu, Sooncheol Kwon, Soo-Young Jang, Seok Kim, Soyeong Jeong, Soonil Hong, Seunghwan Byun, Soon-Ki Kwon, Yun-Hi Kim* and Kwanghee Lee**

Supporting Information

Direct Observation of Confinement Effects of Semiconducting Polymers in Polymer Blend Electronic Systems

Byoungwook Park, Hongkyu Kang*, Yeon Hee Ha, Jehan Kim, Jong-Hoon Lee, Kilho Yu, Sooncheol Kwon, Soo-Young Jang, Seok Kim, Soyeong Jeong, Soonil Hong, Seunghwan Byun, Soon-Ki Kwon, Yun-Hi Kim* and Kwanghee Lee*

This PDF file includes:

Materials and Methods
Figure S1 to S6
Table S1 to S3
Supplementary Reference

Materials and Methods

SP:PS blend solution

Poly(3-hexylthiophene) (P3HT, M_w ~ 53 kg mol^{-1}, Rieke Metals), poly[(E)-1,2-(3,3′-dioctadecyl-2,2′-dithienyl) ethylene-alt-dithieno-(3,2-b:2′,3′-d)thiophene] (P18, M_w ~ 53 kg mol^{-1}, Synthesized)[1], DT-PDPP2T-TT (DPP2T, M_w ~ 73 kg mol^{-1}, 1-Materials),[2] poly[2,5-bis(2-decylnonadecyl)pyrrolo[3,4-c]pyrrole-1,4-(2H,5H)-dione-(E)-1,2-di(2,2′-bithiophen-5yl)ethene] (29-DPP, M_w ~ 61 kg mol^{-1}, Synthesized)[3], regioregular poly[4-(4,4-dihexadecyl-4H-cyclopenta[1,2-b:5,4-b′]dithiophen-2yl)-alt-[1,2,5]thiadiazolo[3,4-c]pyridine] (PCDTPT, M_w ~ 76 kg mol^{-1}, 1-Materials),[4] and Polystyrene (PS, M_w ~ 95 kg mol^{-1}, Sigma Aldrich) were precisely weighed at various weight ratios. Pure semiconducting polymers (SPs) and SPs:PS was dissolved in dehydrated chlorobenzene (P3HT, P3HT:PS, P18, P18:PS, 29-DPP, 29-DPP:PS, PCDTPT and PCDTPT:PS) or dehydrated 1,2,4-trichlorobenzene:chloroform (80:20 volume % ratio) co-solvent (DPP2T and DPP2T:PS) at a fixed total concentration of 2 mg ml^{-1}.

Polymer blend OFET devices fabrication

P3HT:PS, P18:PS, DPP2T:PS and PCDTPT:PS blend OFETs with TGBC configuration: Glass slides (Eagle XGTM, Corning) were cleaned by sequential ultra-sonication in water, acetone, and isopropyl alcohol for least 20 min each. The thermally evaporated Au (30 nm) or Ni/Au (5nm/15nm for P18:PS) source-drain electrodes was patterned using shadow masks. UV/ozone treatment for 20 min was used to clean the surface of substrates. The blending solution was spin-cast onto the substrate in a nitrogen inert atmosphere, and the films were subsequently annealed at 150 ºC for 10 min (P3HT:PS) or 250 ºC for 20 min (P18:PS) or 80 ºC for 10 min (DPP2T:PS) or 200 ºC for 5 min (PCDTPT:PS) to remove residual solvents and optimizing annealing effect. The thickness of the semiconducting layers was controlled to be

~10 to 30 nm. For the gate insulating layer, CYTOP (CTL-809M, Asahi Glass Co., Ltd.) diluted with CT-Solv.180 solvent (4:1 volume ratio) was used. The insulating materials were spin-cast onto the semiconducting layer, resulting in thicknesses of ~ 600 nm, and the films were subsequently annealed at 100 ºC for 10 min. The measured capacitances of CYTOP layers were ~ 3.1 nF cm^{-2}. The devices were finalized by thermal deposition of 50 nm of Al through a shadow mask for the top-gate electrode.

29-DPP:PS blend OFETs with BGTC configuration: Heavily n-doped Si wafer covered with SiO_2 (300 nm) were used as the gate electrode and gate dielectric layer. The substrates were cleaned with acetone, and isopropyl alcohol for least 20 min each. Prior to the polymer deposition, the surface passivation was done by submersion into the 1% solution of ODTS (n-octadecyltrichlorosilane, Sigma Aldrich) in dehydrated toluene (Sigma Aldrich), which was held in a pre-cleaned glass Petri dish at room temperature for 6 hr in a glove box under nitrogen inert atmosphere. The passivated substrates were completely rinsed with toluene. The blending solution was spin-cast onto the substrate in a nitrogen inert atmosphere and the films were subsequently annealed at 200 ºC for 10 min. The thickness of the semiconducting layers was controlled to be ~ 20 nm. The thermally evaporated Au (30 nm) source-drain electrodes were patterned using shadow masks on top of semiconducting layers.

Transparent OFET devices fabrication

Glass/ITO substrate covered with SiO_2 (300 nm, C_i ~ 11.5 nF cm^{-2}.) was used as the gate electrode and gate dielectric layer. Glass/ITO/SiO_2 was cleaned by sequential ultra-sonication in water, acetone, and isopropyl alcohol for least 20 min each. Prior to the polymer deposition, the surface passivation was done by submersion into the 1% solution of ODTS in dehydrated toluene, which was held in a pre-cleaned glass Petri dish at room temperature for 6 hr in a glove box under nitrogen inert atmosphere. The passivated substrates were completely rinsed with toluene. The 29-DPP:PS blending solution was spin-cast onto the substrate in a nitrogen inert

atmosphere and the films were subsequently annealed at 200 ºC for 10 min. The thickness of the semiconducting layers was controlled to be ~ 20 nm. The optimized semitransparent MoO_x (5 nm)/Au (4 nm)/MoO_x (30 nm) source-drain electrode was thermally evaporated on top of semiconducting layer using shadow masks in vacuum chamber with a pressure of 10^{-6} Torr.

Polymer blend OFETs characterization

The *I*-*V* characteristics of the polymer blend OFETs were measured using a Keithley 4200 source meter under nitrogen inert atmosphere. The saturation field-effect mobility was calculated by using the equation $I_{DS} = (WC_i/2L)\mu_{sat}(V_G-V_T)^2$ and the linear field-effect mobility was calculated by using the equation $I_{DS} = (WC_i/L)\mu_{lin}(V_G-V_T)$, where I_{DS} is the drain-source current, μ is the field-effect mobility, W is the channel width, L is the channel length, C_i is the capacitance per unit area of the gate dielectric layer. V_G is the gate voltage, and V_T is the threshold voltage. Since the average thickness of the SP:PS blend layer is than less 30 nm, the corresponding capacitance (including 600nm CYTOP and 300nm SiO_2) is around 3.1 nF cm^{-2} and 11.5 nF cm^{-2}. And, it is surmised that there is a decrease in the effective channel width (W ~ 1000μm) of SP:PS blend films due to the lateral phase separation. To simplify the comparison of the charge transport characteristics, the mobilities of SP:IP blend OFET devices were calculated based on the capacitance of ~ 3.1 n nF cm^{-2} (for CYTOP) and ~ 11.5 nF cm^{-2} (for SiO_2) and the same channel width of the pure SP films. Though the simplification of calculation of charge carrier mobility leads to a slightly underestimation, there is no influence on key conclusions of this work.

In addition, it is presumed that there is a decrease in the effective channel width (W ~ 1000μm) of SP:PS blend films due to the lateral phase separation, the charge carrier mobility was extracted as the same channel width of the pure SP films.

AFM characterization

The topographic images of the samples were collected by AFM in the tapping mode to probe the differences in the surface feature of the semiconducting polymer films

TEM characterization

The samples were obtained by peeling the spin-casted films, which were subsequently transferred onto 200 mesh copper grids (Electron Microscopy Sciences). Top-down TEM images of the sample films were recorded using a Tecnai G2 F30 S-Twin microscope operated at an acceleration voltage of 300 keV.

X-ray characterization

Grazing-incidence wide-angle X-ray scattering measurements were performed at the 3C-WAXSI beam line in the Pohang Accelerator Laboratory (PAL) using a monochromatic X-ray radiation source of 10.07 keV (λ = 1.230 Å) and a 2D X-ray detector (Mar165 CCD). The samples were placed on a z-axis goniometer and were maintained under vacuum conditions (~10^{-3} Torr) during irradiation.

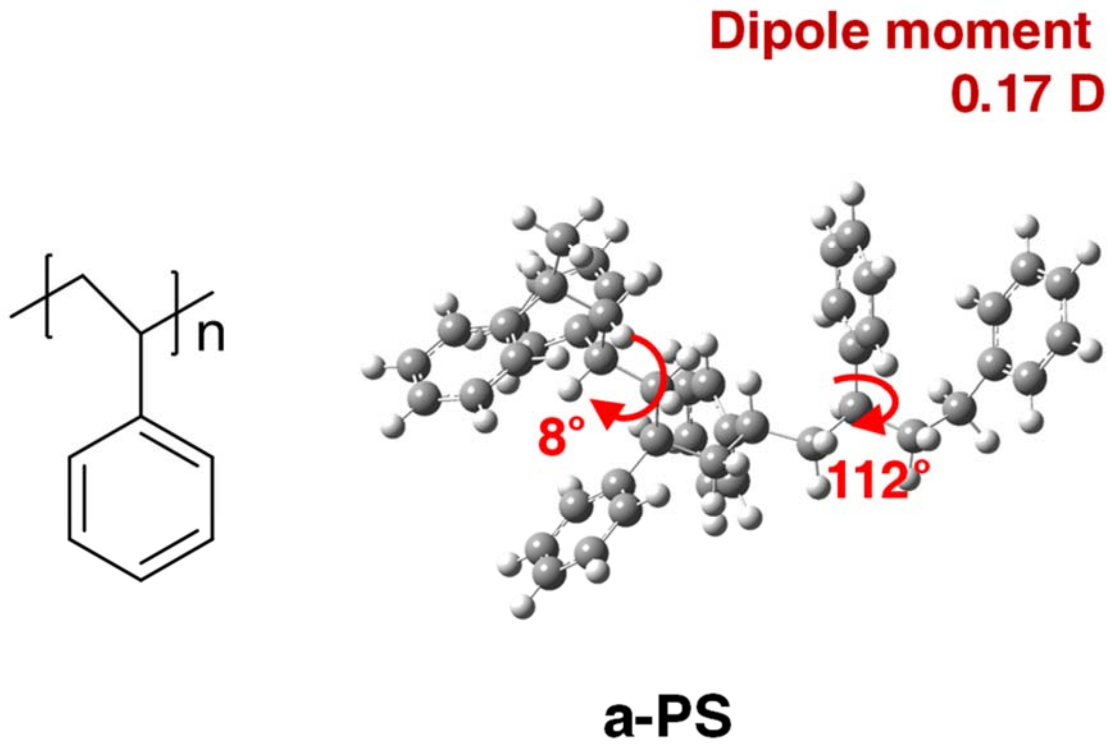

Figure S1. DFT-optimized molecular models and calculated dipole moments of the tetramer of atactic polystyrene (a-PS).

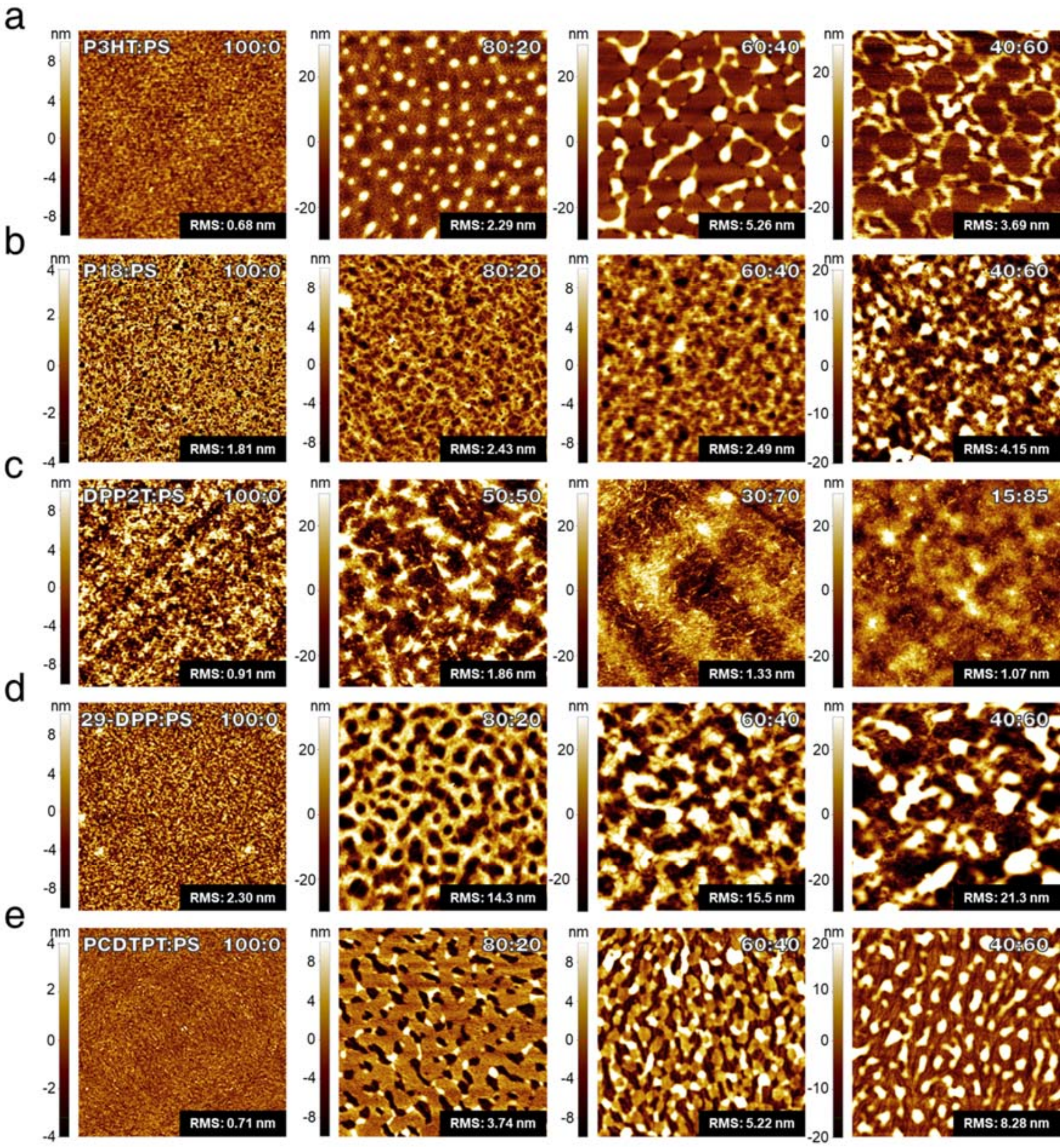

Figure S2. Morphological characteristics of SP:PS blend films. 2D top-surface images (5 μm x 5 μm) of (a) P3HT:PS (100:0 ~ 40:60), (b) P18:PS (100:0 ~ 40:60), (c) DPP2T:PS (100:0 ~ 15:85), (b) 29-DPP:PS (100:0 ~ 40:60) and (e) PCDTPT:PS (100:0 ~ 40:60) blend films measured using atomic force microscopy (AFM) in the tapping mode.

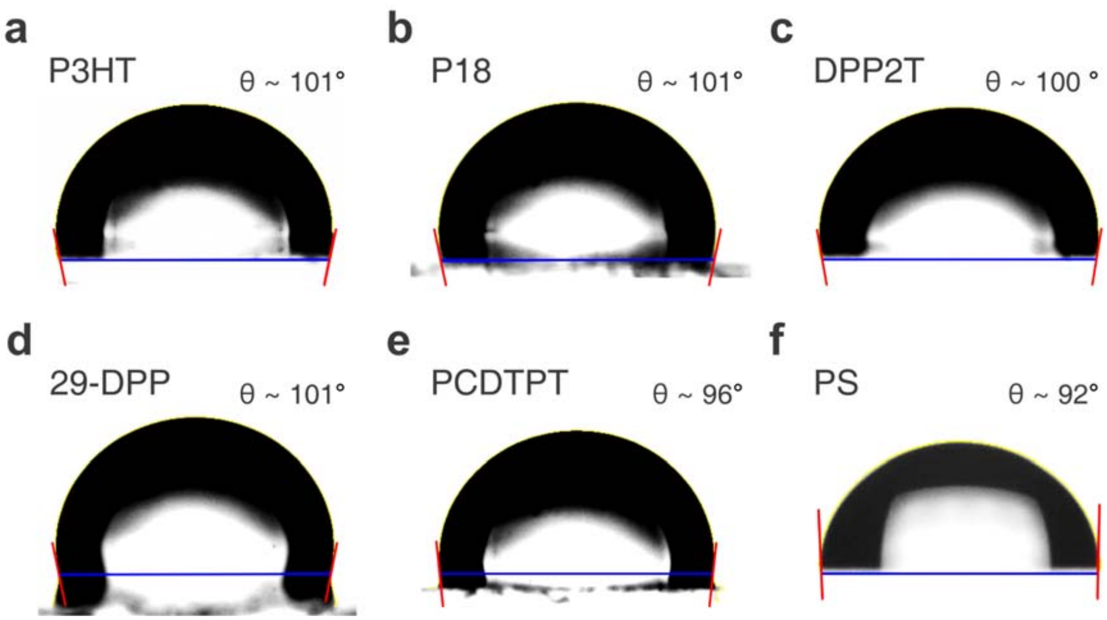

Figure S3. Water contact angles measured on (a) P3HT, (b) P18, (c) DPP2T, (d) 29-DPP, (e) PCDTPT and (f) PS films.

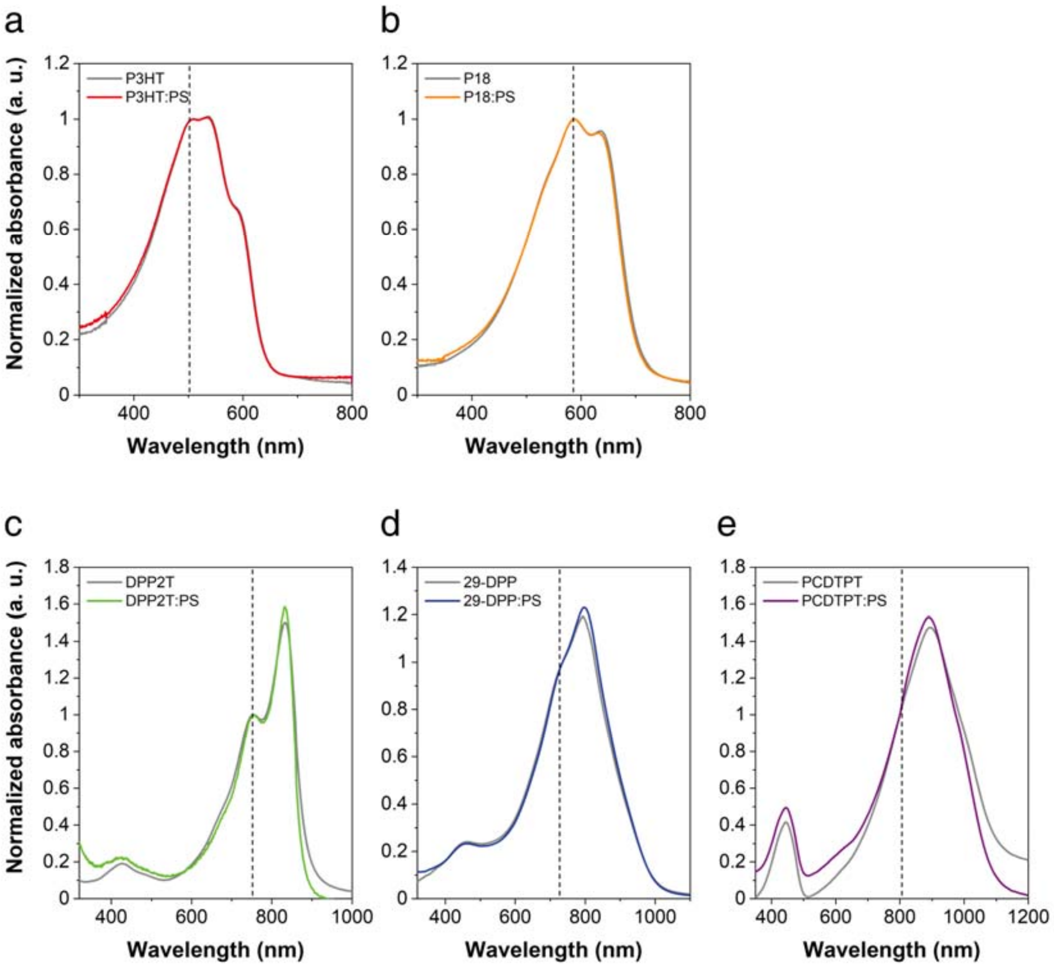

Figure S4. Comparison of absorption characteristics of pure SP and SP:PS blend films. Absorption spectra of (a) P3HT:PS, (b) P18:PS, (c) DPP2T:PS, (d) 29-DPP:PS, (e) PCDTPT:PS films.

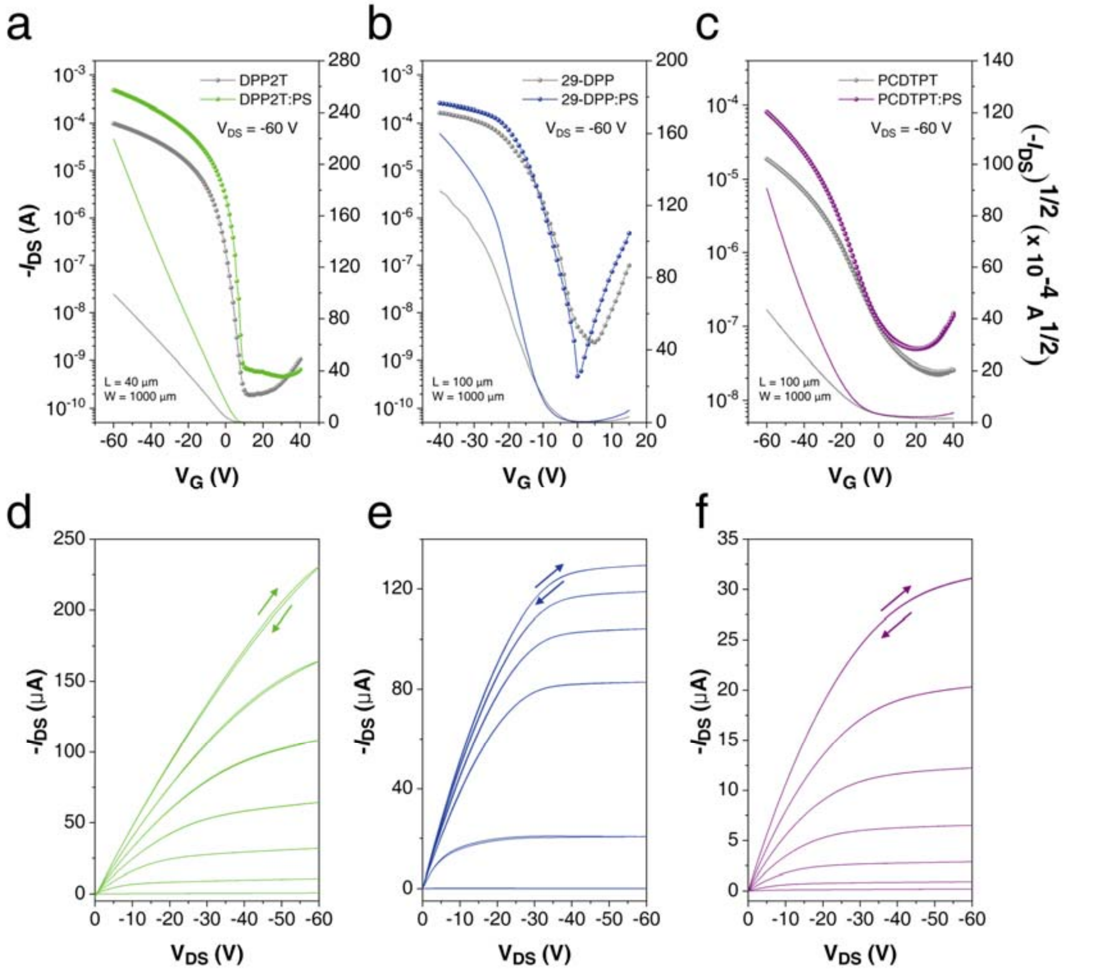

Figure S5. Transfer and Output characteristics of polymer blend OFETs at room temperature. Transfer curve of (a) DPP2T, DPP2T:PS blend, (b) 29-DPP, 29-DPP:PS blend, (c) PCDTPT and PCDTPT:PS blend OFETs. Output curve of (d) DPP2T:PS, (e) 29-DPP2T:PS and (f) PCDTPT:PS blend OFETs. V_{GS} varies from 0 V to -60 V.

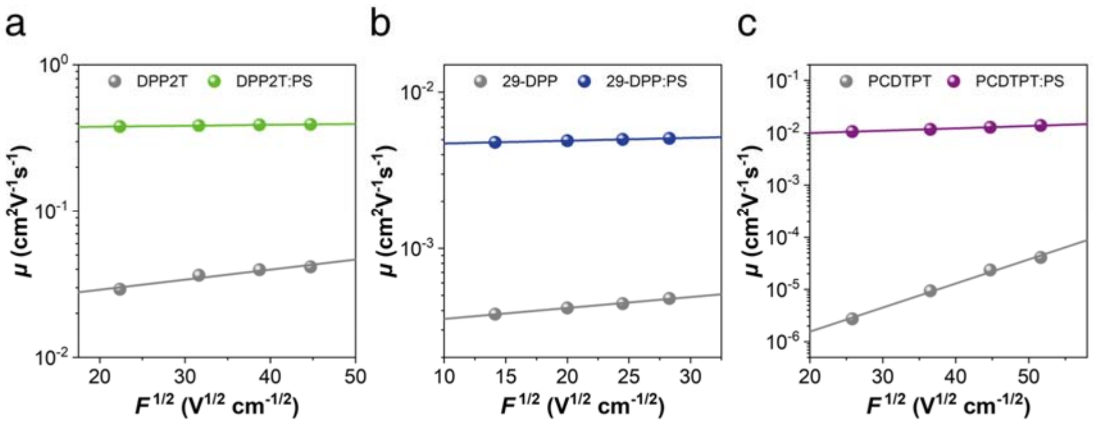

Figure S6. Electric-field (F) dependence characteristics of the pure SPs and SP:PS blend films at a low temperature of 100 K. Plot of the linear hole mobility as a function of applied electric field ($F^{1/2}$) for (a) DPP2T, DPP2T:PS blend, (b) 29-DPP, 29-DPP:PS blend, (c) PCDTPT and PCDTPT:PS blend OFETs at T = 100 K.

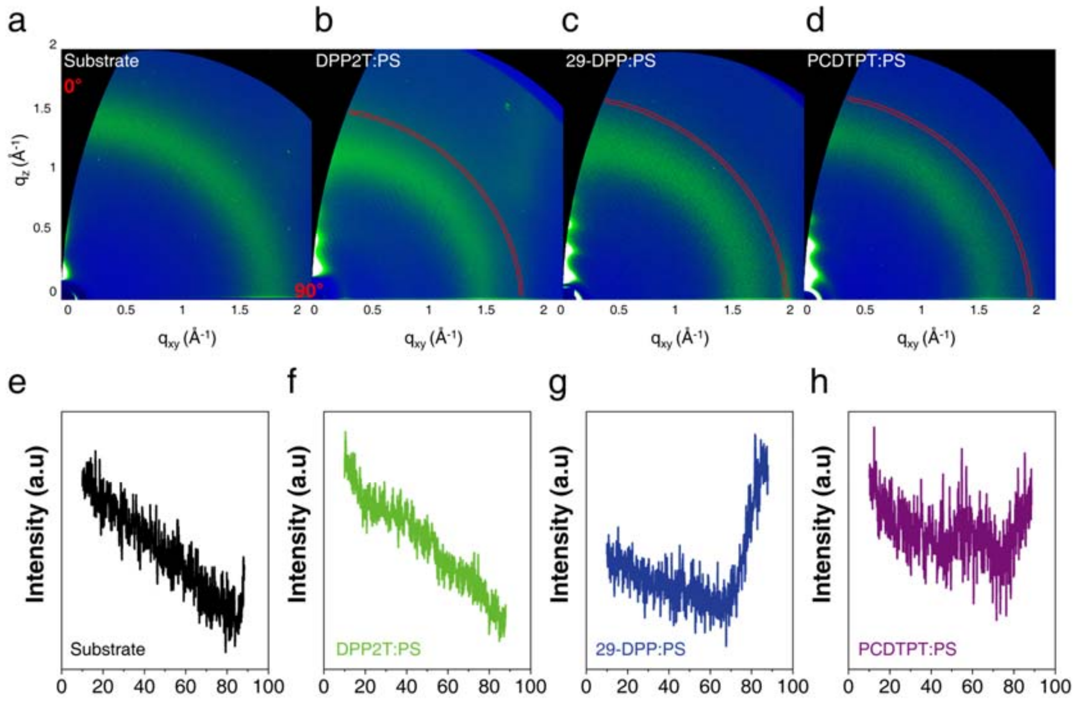

Figure S7. Azimuthal intensity plots of the (010) peaks of the SP:PS blend films. 2D GIWAXS images recorded for (a) the Si/SiO2 substrate, (b) the DPP2T:PS blend film, (c) the 29-DPP:PS blend film and (d) the PCDTPT:PS blend film. The corresponding 1D azimuthal intensity profiles of the amorphous halo peak of (e) the Si/SiO2 substrate and the (010) peak of (f) the DPP2T:PS blend film, (g) the 29-DPP:PS blend film and (h) the PCDTPT:PS blend film.

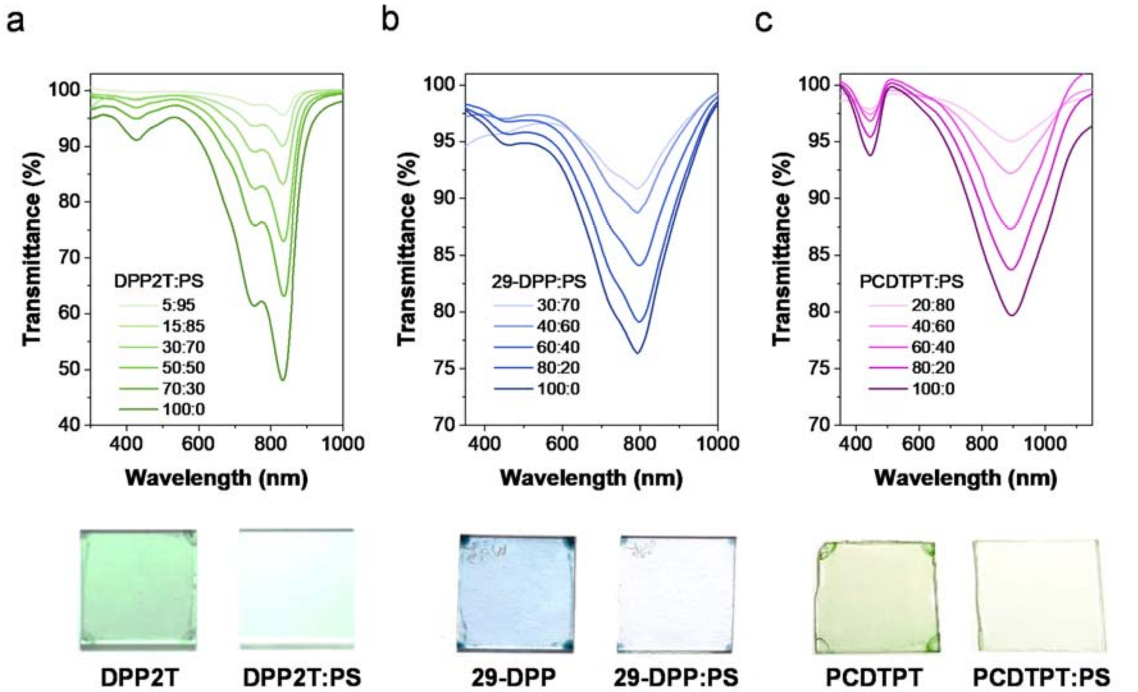

Figure S8. Comparison of transmittance characteristics of pure SP and SP:PS blend films. Transmittance spectra of (a) DPP2T:PS, (b) 29-DPP:PS, (c) PCDTPT:PS films at various concentration ratios and optical images of pure SPs and SP:PS blend films on glass substrates.

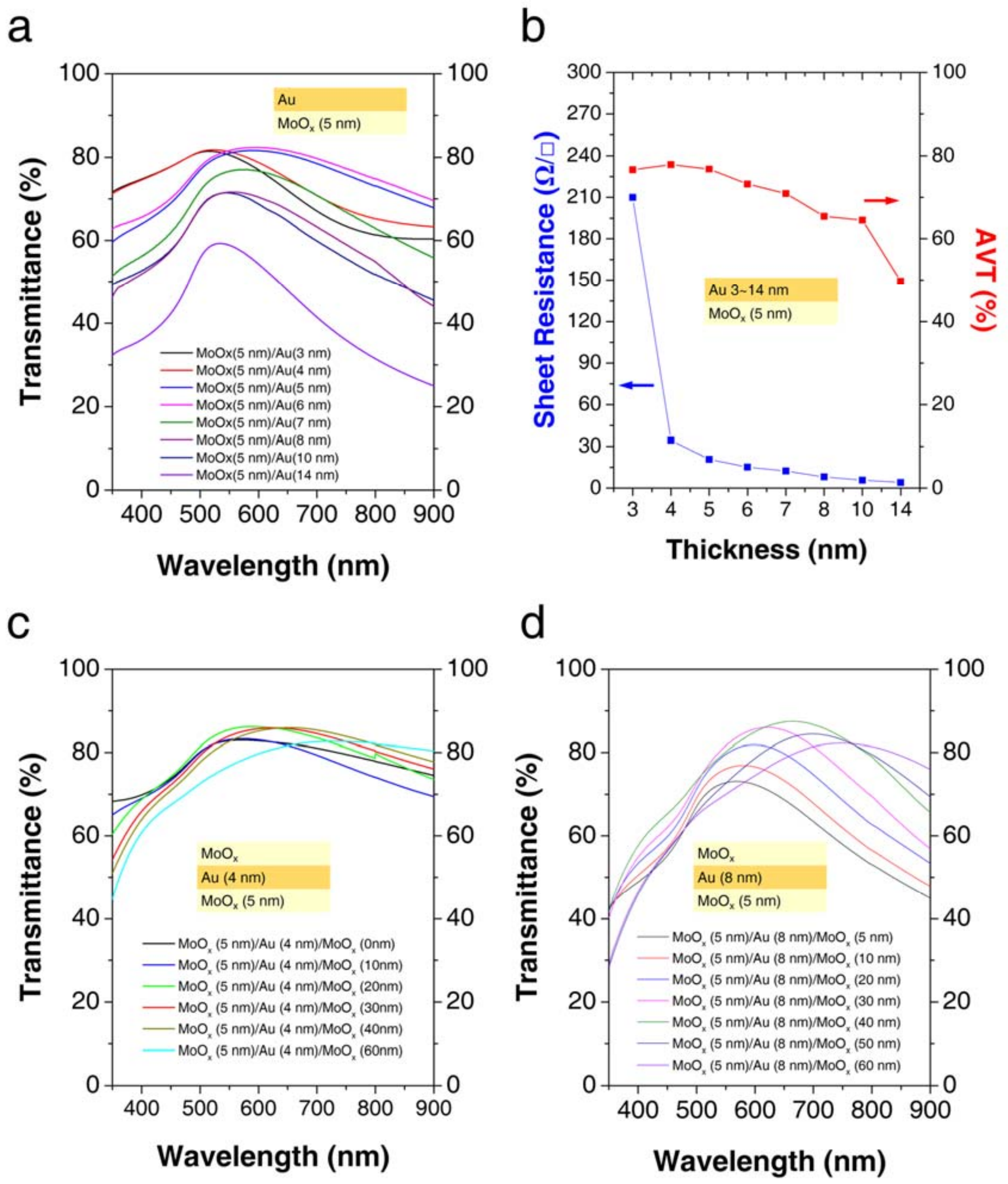

Figure S9. Optimization of metal oxide/metal/metal oxide (OMO) transparent electrode. (a) Transmittance spectra of molybdenum oxide (MoO_x)/gold electrode (Au) with different thickness of Au. (b) Plot of sheet resistance and average visible light transmittance (AVT) according to decrease of thickness of Au. Transmittance spectra of (c) MoO_x (5 nm)/ Au (4 nm) or (d) MoO_x (5 nm)/ Au (8 nm) with different thickness of top MoO_x.

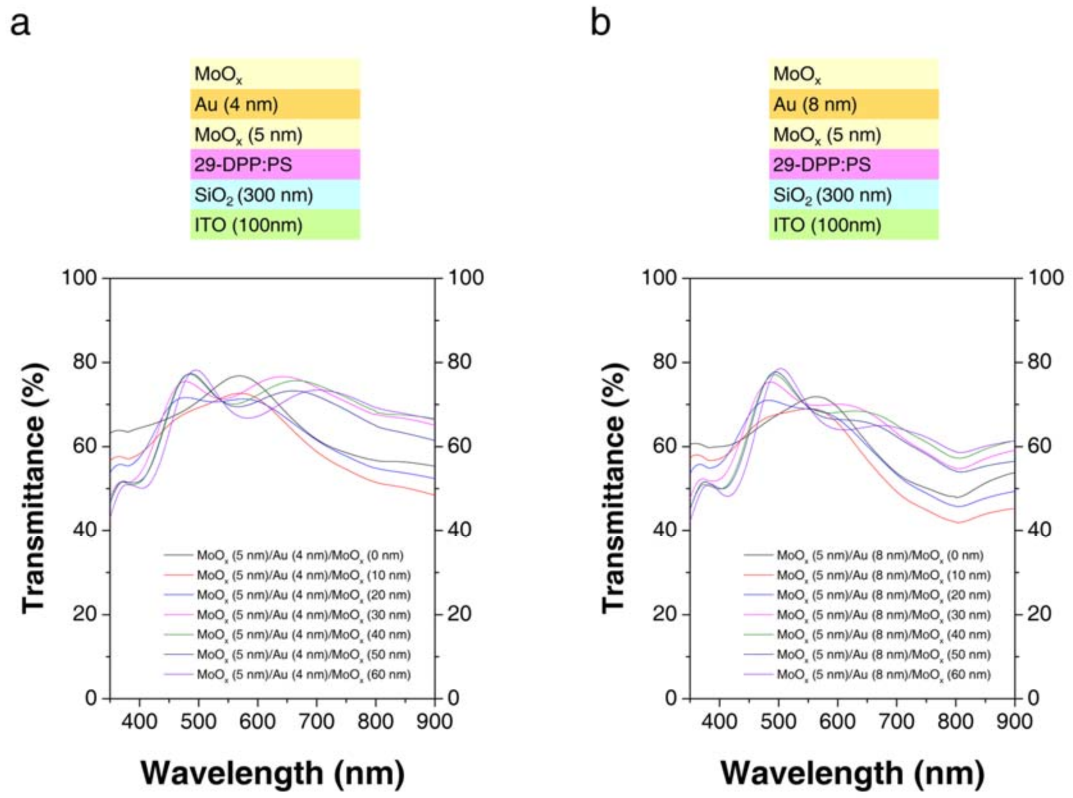

Figure S10. Optimization of transmittance of all transparent 29-DPP:PS blend OFETs. Transmittance spectra of (a) ITO (100 nm)/SiO2 (300 nm)/29-DPP:PS (AVT > 95%)/MoO_x (5 nm)/Au (4 nm) or (b) ITO (100 nm)/SiO2 (300 nm)/29-DPP:PS (AVT > 95%)/MoO_x (5 nm)/Au (8 nm) with different thickness of top MoO_x from 0 to 60 nm.

Table S1. Water contact angles and caculated surface energies of P3HT, P18, DPP2T, 29-DPP, PCDTPT and PS films.

Materials	Semiconducting Polymer					Insulating Polymer
	P3HT	P18	DPP2T	29-DPP	PCDTPT	PS
Contact Angle (°)	101	101	100	101	96	92
Surface Energy (mN/m)	11.8	11.8	12.3	11.8	14.4	16.8

Table S2. Mobility values of the P3HT:PS and P18:PS OFETs with various concentration ratios[a]

	P3HT:PS			P18:PS	
P3HT Contents (%)	μ_a ($cm^2 V^{-1} s^{-1}$)	μ_{max} ($cm^2 V^{-1} s^{-1}$)	P18 Contents (%)	μ_a ($cm^2 V^{-1} s^{-1}$)	μ_{max} ($cm^2 V^{-1} s^{-1}$)
100	$5.28 \times 10^{-2} \pm 2.00 \times 10-2$	7.44×10^{-2}	100	$2.66 \times 10^{-1} \pm 1.90 \times 10-2$	2.86×10^{-1}
90	$4.15 \times 10^{-2} \pm 1.42 \times 10-2$	6.72×10^{-2}	90	$2.20 \times 10^{-1} \pm 3.61 \times 10-2$	2.46×10^{-1}
70	$6.18 \times 10^{-2} \pm 1.91 \times 10-2$	8.14×10^{-2}	70	$1.78 \times 10^{-1} \pm 2.21 \times 10-2$	1.99×10^{-1}
50	$2.11 \times 10^{-2} \pm 1.16 \times 10-2$	3.10×10^{-2}	50	$8.10 \times 10^{-2} \pm 1.41 \times 10-2$	9.95×10^{-2}
30	$2.35 \times 10^{-2} \pm 2.74 \times 10-3$	2.64×10^{-2}	30	$2.77 \times 10^{-3} \pm 8.06 \times 10-4$	3.34×10^{-3}
10	$1.49 \times 10^{-4} \pm 9.51 \times 10-5$	2.58×10^{-4}	10	$8.01 \times 10^{-5} \pm 3.94 \times 10-5$	1.36×10^{-4}

μ_a and μ_{max} is the average and maximum mobilities, respectively.
[a] Mobility values were summarized from more than 5 devices for each concentration ratios.

Table S3. Device performances of the DPP2T:PS, 29-DPP:PS and PCDTPT:PS OFETs with various concentration ratios[a]

	DPP2T:PS		
DPP2T Contents (%)	μ_a ($cm^2 V^{-1} s^{-1}$)	μ_{max} ($cm^2 V^{-1} s^{-1}$)	I_{on}/I_{off}
100	0.69 ± 0.09	0.91	$> 10^5$
80	0.85 ± 0.08	0.98	$> 10^5$
50	1.08 ± 0.22	1.49	$> 10^5$
20	1.13 ± 0.30	1.60	$> 10^5$
15	1.49 ± 0.47	3.10	$> 10^5$
10	1.14 ± 0.28	1.78	$> 10^5$
5	0.87 ± 0.23	1.29	$> 10^5$
	29-DPP:PS		
29-DPP Contents (%)	μ_a ($cm^2 V^{-1} s^{-1}$)	μ_{max} ($cm^2 V^{-1} s^{-1}$)	I_{on}/I_{off}
100	4.23 ± 0.24	4.74	$> 10^5$
80	6.78 ± 1.11	8.87	$> 10^5$
70	8.12 ± 1.17	9.39	$> 10^6$
60	8.33 ± 0.50	8.73	$> 10^6$
50	7.91 ± 1.00	10.2	$> 10^6$
40	5.59 ± 1.48	7.30	$> 10^5$
20	1.05 ± 0.93	2.49	$> 10^4$
	PCDTPT:PS		
PCDTPT Contents (%)	μ_a ($cm^2 V^{-1} s^{-1}$)	μ_{max} ($cm^2 V^{-1} s^{-1}$)	I_{on}/I_{off}
100	0.28 ± 0.14	0.61	$> 10^2$
80	0.65 ± 0.17	1.04	$> 10^2$
60	0.63 ± 0.24	1.22	$> 10^3$
40	0.48 ± 0.08	0.65	$> 10^2$
20	0.23 ± 0.07	0.39	$> 10^2$
15	0.09 ± 0.07	0.23	$> 10^2$
10	0.02 ± 0.03	0.10	$> 10^2$

μ_a and μ_{max} is the average and maximum mobilities, respectively.
[a] Mobility values were summarized from more than 10 devices for each concentration ratios.

Table S4. Transmittance values of all transparent 29-DPP:PS blend OFET device with different layering conditions.

Condition	T_a (%)	T_{max} (%)	T_{min} (%)	T_{550} (%)
ITO/SiO_2	94.29	100	86.10	95.40
ITO/SiO_2/29-DPP:PS	89.68	93.93	84.21	88.74
ITO/SiO_2/29-DPP:PS/MoO_x/Au/MoO_x	72.13	76.64	53.44	71.50

T_a, T_{max} and T_{min} are the average, the maximum and the minimum transmittances, respectively. T_{550} is the transmittance at wavelength 550 nm. These values were estimated in the visible range (400 nm ~ 700 nm).

Supplementary References

[1] S.-Y. Jang, I.-B. Kim, J. Kim, D. Khim, E. Jung, B. Kang, B. Lim, Y.-A. Kim, Y. H. Jang, K. Cho, *Chem. Mater.* **2014**, *26*, 6907.

[2] W. Li, K. H. Hendriks, W. S. C. Roelofs, Y. Kim, M. M. Wienk, R. A. J. Janssen, *Adv. Mater.* **2013**, *25*, 3182.

[3] I. Kang, H. J. Yun, D. S. Chung, S. K. Kwon, Y. H. Kim, *J. Am. Chem. Soc.* **2013**, *135*, 14896.

[4] L. Ying, B. B. Y. Hsu, H. Zhan, G. C. Welch, P. Zalar, L. A. Perez, E. J. Kramer, T. Q. Nguyen, A. J. Heeger, W. Y. Wong, G. C. Bazan, *J. Am. Chem. Soc.* **2011**, *133*, 18538.
